# Thermophysical Properties of Vegetable Oil-Based Hybrid Nanofluids Containing Al_2_O_3_-TiO_2_ Nanoparticles as Insulation Oil for Power Transformers

**DOI:** 10.3390/nano12203621

**Published:** 2022-10-15

**Authors:** Vignesh Vicki Wanatasanappan, Munirah Rezman, Mohd Zulkifly Abdullah

**Affiliations:** 1 Institute of Power Engineering, Universiti Tenaga Nasional, Kampus Putrajaya, Kajang 43000, Malaysia; 2 College of Engineering, Universiti Tenaga Nasional, Kampus Putrajaya, Kajang 43000, Malaysia; 3 School of Mechanical Engineering, Engineering Campus, Universiti Sains Malaysia, Pulau Pinang 14300, Malaysia

**Keywords:** vegetable oil, hybrid nanofluid, transformer oil, Al_2_O_3_-TiO_2_, density, thermal conductivity, viscosity

## Abstract

The massive demand in the electrical power sector has resulted in a large demand for reliable, cost efficient, and environmentally friendly insulation oil to reduce the dependency on mineral oil. The hybridization of nanoparticles in vegetable oil is a novel method to enhance the thermal properties of vegetable oil. This study focuses on the experimental investigation of the thermophysical properties of coconut oil, soybean oil, and palm oil-based hybrid nanofluids suspended with Al_2_O_3_-TiO_2_ nanoparticles at a mass concentration of 0.2, 0.4, and 0.6%. The ratio between Al_2_O_3_ and TiO_2_ nanoparticles was maintained constant at 50:50. The main purpose of the study is to evaluate the thermal conductivity, dynamic viscosity, and density of different vegetable base oils suspended with Al_2_O_3_-TiO_2_ in the temperature range of 30 to 60 °C. The influence of temperature on the augmentation of thermophysical properties for different vegetable oil-based hybrid nanofluids is investigated experimentally. The experimental results for thermal conductivity for the three types of base fluids show that the effect of nanoparticle mass concentration in thermal conductivity enhancement is less significant for temperatures more than 50 °C. The palm oil with a 0.6% Al_2_O_3_-TiO_2_ nanoparticle concentration exhibited the highest thermal conductivity with a 27.5% thermal conductivity enhancement relative to the base oil. The effect of nanofluid temperature on density and viscosity augmentation is more distinct compared with the impact of Al_2_O_3_-TiO_2_ nanoparticles concentrations. Among all three types of hybrid nanofluids, palm oil based nanofluids were found to have superior thermophysical properties compared with coconut oil and soybean oil, with the highest thermal conductivity of 0.628 W/m·k and lowest viscosity of 17.772 mPa·s.

## 1. Introduction

Mineral oil is used in various applications for lubrication, as a cutting fluid, coolant, and for insulation. One of the prime applications of mineral oil is for insulation and cooling purposes in power transformers [1]. For decades, mineral oils such as naphthenic oil have been used in power transformers due to their low cost, good thermal capacity, and good pouring point at low temperatures. However, mineral oil has several limitations that make it less sustainable for future generations. For example, naphthenic oil is extracted from a non-renewable source, and it has a high fire risk and low biodegradability [2]. Other fluids, such as ester dielectric fluid, have been proposed but did not gain much popularity due to their high cost and poor biodegradability. Therefore, a new ecological solution is needed to replace the dependency on mineral oil for insulation and cooling purposes.

Various research work has been carried out around the world to find an alternative solution to replace mineral oil as insulating oil in power transformers [3,4,5,6]. For example, Fontes et al. [4] evaluated the thermal conductivity, viscosity and breakdown voltage of transformer oil based nanofluid consisting of carbon nanotubes and diamond nanoparticles. They used multi-walled carbon nanotubes (MWCNT) and diamonds with a size of 5 nm and 6 nm, respectively. A two-step method was applied to disperse the nanoparticles in mineral oil (MO), which acts as base fluid. According to their experimental results, thermal conductivity enhancement using MWCNT is higher than when using diamond-based mineral oil nanofluid. However, their results on dynamic viscosity revealed that the diamond-based nanofluid has a lower viscosity, and the dielectric strength of the nanofluid was reduced by almost half compared with mineral oil without any suspension. Another study by Ghasemi et al. [7] focused on analyzing the breakdown voltage of transformer oil suspended with Fe_3_O_4_ nanoparticles at a volume concentration between 0.1 and 0.6%. The authors found that the lightning impulse breakdown voltage increases due to the magnetic and dielectric properties of Fe_3_O_4_ nanoparticles in the transformer oil. Recently, Cimbala et al. [8] evaluated the dielectric response of transformer oil filled with iron oxide and fullerene nanoparticles. Their study revealed that the transformer oil-based nanofluids with 0.01% *w*/*v* concentration achieved maximum dielectric relaxation close to an ideal shape. Most past research focuses mainly on improving the thermal and dielectric properties of mineral oil using nanoparticles. To date, the theories available only explain the increase in breakdown voltage of transformer based nanofluids. However, no single theory is focused on the thermophysical properties such as density, thermal conductivity, and dynamic viscosity. Even though nanoparticles have a high potential to improve these properties, it does not solve the dependency on mineral oil and work toward an alternative oil. One of the potential alternatives to mineral oil is vegetable oil.

Vegetable oil consists of triglycerides extracted from plants or seeds [9,10]. Vegetable oil is extracted mainly from seeds, nuts, cereals, grains and fruits. These oils or fats have been used for cooking purposes for much of human history. Some examples of vegetable oils are soybean oil, coconut oil, punna oil, rice bran oil, palm oil, gingelly oil, sunflower oil, neem oil, castor oil, mustard oil, ground nut oil and mughwa oil [11,12,13]. Vegetable oil tends to be a suitable alternative due to its sustainability and properties, such as being biodegradable, environmentally friendly, inexpensive, and having a high flash point [14,15]. In contrast to mineral oil, vegetable oil such as coconut oil is non-toxic and is known to be an environmentally friendly dielectric liquid [16]. The issue of soil contamination from waste transformer oil (WTO) can be avoided with the use of natural ester-based substances such as vegetable oil. In a recent report by Tiwari et al. [17] on soil contamination caused by WTO, they highlighted the presence of heavy metals in WTO that affects the microbiological activity of soil and the adverse effects on human beings and other living things. However, one of the significant limitations of vegetable oil is the high viscosity value which limits the heat dissipation capacity.

Hui Yu et al. [18] investigated low density dielectrics based on vegetable oil methyl esters through a transesterification and purification process. They evaluated the electrical and physical properties of methyl ester synthesized from soybean oil, canola oil, peanut oil and cottonseed oil. They found the breakdown voltage of methyl esters to be higher than that of mineral oil, which is around 49 kV. However, the authors observed a major weakness in methyl esters extracted from vegetable oil, namely, the poor low temperature fluidity characterized by a high pour point. In a separate study, Deraman et al. [14] evaluated the potential application of waste cooking oil (WCO) experimentally as insulation oil in transformers. Since WCO has high acidity, high water content, and a low breakdown voltage, the authors performed a transesterification procedure and then post treatment to the methyl esters. They could reduce the acidity of WCO by 90.78%; however, they could still not meet the acidity target for the insulating oil of 0.06 mg KOH/g. Nevertheless, their results for the breakdown voltage and water content were found to improve significantly. 

Rojas et al. [15] researched the thermophysical properties of four different vegetable oils including soybean oil, cotton oil, canola oil and sunflower oil. They carried out the experiments at 15 different temperature intervals between 26 and 160 °C. The measured data were analyzed using the statistical tool SAS. The authors concluded that the temperature variation affected the thermophysical properties of all vegetable oil tested. The predictive model developed to estimate dynamic viscosity had a coefficient of determination (R^2^) value of 0.83. Meanwhile, the linear model developed to predict the values had a good correlation with the experimental data for the density, heat capacity and thermal conductivity. Katim et al. [19] experimented with rapeseed oil (RO) as a potential replacement for mineral oil used in transformers. The researchers synthesized RO and measured the AC breakdown voltage, dissipation factor, and resistivity according to IEC 60156 and IEC 60247 standards and various temperature conditions. They achieved a comparable breakdown voltage of RO with mineral oil. However, the dissipation factor increased highly with temperature which was the primary concern highlighted by the study. Based on a feasibility study conducted by Leyla et al. [20], the thermophysical properties of waste cooking oil (WCO) demonstrated an almost significant cooling performance compared with mineral oil. However, further enhancement is required to use vegetable oil as a pure substitute for mineral oil, especially in relation to thermophysical properties. Some possible methods to address this gap are nanotechnology and hybridization techniques.

The revolution in nanotechnology has been able to improve the properties of vegetable oil through the inclusion of nanoparticles. Nanofluid is known as a new generation fluid which is developed by suspension of solid particles typically sized less than 100 nm in any base fluid. This technology has been researched for various cooling applications such as automobile radiators, solar panel cooling, battery cooling in electric vehicles, biomedical technology and coolant in the machining process [21,22,23]. For the application of oil as transformer insulation, only mono nanofluid has been tested in vegetable oil. This was tested by Jian li et al. [24], investigating the breakdown voltage and dielectric properties of rapeseed oil suspended with Fe_3_O_4_. Interestingly, the suspension of 0.004% weight percentage of Fe_3_O_4_ in RO enhanced the breakdown voltage of oil based nanofluid by 20% compared with the RO. Their research also demonstrated that the relative permittivity of Fe_3_O_4_/RO-based nanofluid is significantly higher than the base oil itself. The suspension of nanoparticles in a base fluid such as vegetable oil using the ultrasonication technique is able to produce oil-based nanofluid. Mengata et al. [25] researched the effect of FeO_3_ nanosized particles on the thermodynamic and physicochemical properties of kernel palm oil methyl ester (KPOME). They measured the thermal conductivity of nanofluid and observed that the suspension of nanoparticles at 0.2 wt.% recorded the maximum thermal conductivity value for temperatures between 40 and 65 °C. In addition, the authors observed an increase in the kinematic viscosity with an increase in the nanoparticle concentration. Although there is much research on mono vegetable oil based nanofluids, the major challenge that still remains is improving the thermophysical properties and stability. One way to overcome this limitation is using the hybridization technique to develop hybrid nanofluids.

Hybrid nanofluids are the suspension of two or more types of nanoparticles in the base fluid to impart the property of different materials in the base fluid Significant research works are available on water or ethylene glycol (EG)-based hybrid nanofluids. It is known that such hybrid nanofluids have potential applications in heat transfer mechanisms such as heat exchangers, heat pipes, micro/mini channel cooling, solar panel cooling and electric vehicle battery cooling. However, the research work on vegetable oil-based hybrid nanofluids has not been well explored. Many researchers have investigated and reported the positive influence of nanoparticles such as Al_2_O_3_ and TiO_2_ in improving the thermophysical properties of base fluid water [10,26,27,28,29,30,31,32]. For example, Chiam et al. [33] evaluated the thermal conductivity and viscosity of Al_2_O_3_ nanofluids using a water–EG mixture at three different ratios of 40:60, 50:50 and 60:40. According to these researchers, the thermal conductivity was enhanced by about 12.8% and the 60:40 water-EG ratios were found to be the optimum. Additionally, the viscosity of the nanofluid in their study almost doubled with the suspension of Al_2_O_3_ nanoparticles. The effects of Al_2_O_3_-TiO_2_ mixture ratios on the thermophysical properties of a water base fluid was specifically tested by Vicki et al. [34]. The authors concluded an average thermal conductivity augmentation of 71% for Al_2_O_3_-TiO_2_ ratio of 50:50 with a liquid temperature of 70 °C. Moreover, they also found that dispersingAl_2_O_3_-TiO_2_ nanoparticles at various ratios in water does not change the Newtonian fluid characteristic of the base fluid. In a very recent study by Jacek et al. [35] using TiO_2_ nanoparticles in EG base fluid, the researchers found that pure anatase phase TiO_2_ can enhance the electrical properties of EG significantly with 20 wt.% nanoparticles.

Based on the literature, it is well reported that the hybridization of Al_2_O_3_ and TiO_2_ has good potential for thermophysical property improvement. However, the combination of these two nanoparticles as hybrids in vegetable oil is yet to be explored. In addition, the findings on the compatibility of Al_2_O_3_-TiO_2_ in different vegetable oils is valuable for future researchers in the field of transformer insulating oil. The hybridization of Al_2_O_3_-TiO_2_ in vegetable base oils such as coconut oil, soy bean oil and palm oil is an innovative method to improve the thermophysical properties of vegetable oil. The insulation oil used in power transformers not only functions as a dielectric fluid but also works as a cooling agent to maintain the temperature of the components in a transformer. Mono nanofluids that use vegetable oil still have not met the requirements as an alternative for mineral oil in power transformers. In addition, most of the past research is focused mainly on the enhancement of the electrical properties of vegetable oil. For an effective cooling fluid, thermophysical properties such as density, thermal conductivity, and dynamic viscosity play an important role. Therefore, the present study focuses on experimenting with the effect of hybrid Al_2_O_3_-TiO_2_ nanoparticles on the density, thermal conductivity, and dynamic viscosity of vegetable base oil consisting of coconut oil, soybean oil and palm oil. The nanoparticles were suspended in vegetable base oils using a two-step method at concentrations of 0.2–0.6%. The thermophysical properties of these hybrid nanofluid vegetable oils were evaluated experimentally for all base oils. The influence of temperature on the thermophysical properties was investigated for a temperature range between 30 and 45 °C. The outcome of the present research work provides valuable knowledge on vegetable oil-based hybrid nanofluids and the compatibility of vegetable base oils with hybrid Al_2_O_3_-TiO_2_ nanoparticles to be used as insulation oil for transformers.

## 2. Materials and Methods

### 2.1. Materials Used

The present study used three types of vegetable oil: food grade coconut oil, palm oil, and soybean oil. This vegetable oil served as the base fluid for the hybrid nanofluid. The two different nanoparticles Al_2_O_3_ and TiO_2_ were obtained from Sigma Aldrich. In addition, cetyltrimethylammonium bromide (CTAB), which was used as a surfactant, was also supplied by Sigma Aldrich, Saint Louis, MO, USA. The average particle size of the Al_2_O_3_ is reported to be less than 13 nm, and the size of TiO_2_ is less than 21 nm. The properties of the nanoparticles used are shown in Table 1, and the properties of base fluids are listed in Table 2.

### 2.2. Dispersion of Al_2_O_3_ and TiO_2_ Nanoparticles in Base Oils

The dispersion of the nanoparticles in the base fluid involved a two-step method. The first step is the dispersion of Al_2_O_3_-TiO_2_ nanoparticles and surfactant (cetyltrimethylammonium bromide (CTAB) in vegetable oils by a magnetic stirring process for 2 h. The purpose of using CTAB surfactant is to ensure the homogenous dispersion of nanoparticles in the base oils and to produce a stable nanofluid. The use of CTAB in improving stability was demonstrated by Arora et al. [36] in their experimental work on MWCNT-based nanofluid. The second step involved the homogenous dispersion of nanoparticles in base oils using an ultrasonic probe sonicator (Q55 Sonicator, Q Sonica, Newtown, CT USA) for 1.5 h with 55% amplitude. An ice bath was used to prevent overheating of the nanofluid mixture during the probe sonication process. The mixture was also sonicated using an ultrasonic bath (Elmasonic S120H, Singen, Germany) for 3 h to avoid agglomeration of the nanoparticles in the base oils. The ratio between the Al_2_O_3_ and TiO_2_ nanoparticles was constant at 50:50 for all the base oil samples with nanoparticle mass concentrations of 0.2%, 0.4%, and 0.6%. The 50:50 ratio of Al_2_O_3_ and TiO_2_ was selected based on the outcome of the study conducted by Gunnasegaran et al. [34] using water as the base fluid, where the 50:50 nanoparticle composition exhibited the best stability and optimum thermal conductivity value. As for the CTAB, the ratio was about 1:10 of the Al_2_O_3_-TiO_2_ nanoparticle mass ratio. Each sample was prepared using 80 mL of coconut oil, palm oil or soybean oil. The prepared mixture was left undisturbed and observed by visible inspection for a period of one month. The soybean oil and palm oil with Al_2_O_3_-TiO_2_ nanoparticles of 0.2% mass concentration displayed good stability with minimal sedimentation even after one month.

### 2.3. Morphological and Structural Characterization

The XRD characterization technique was used to identify the structure of the nanoparticles based on the diffraction pattern. The intensity of the XRD pattern was recorded using a Shimadzu XRD-6000 diffractometer (Kyoto, Japan) with Cu-Kα radiation over a diffraction angle (2θ) ranging from 15° to 70°, with a step size of 0.02°. The nano crystallite sizes (L) of the Al_2_O_3_ and TiO_2_ particles were calculated using the XRD radiation of wavelength λ (nm) and full width at half maximum of peaks (β) in radian located at any three dominant 2θ peaks. For the calculation, the Scherrer formula (L = Kλ/β·cosθ) was used with the shape factor K kept constant at 0.9. In addition, TEM analysis was performed on the nanofluid to analyze the morphology of the nanoparticles and estimate the size of the nanoparticles for comparison with the XRD data.

### 2.4. Thermal Conductivity Measurement

One of the techniques that has been adopted by many researchers for thermal conductivity measurement of nanofluids is the transient hot-wire method (THW), a steady-state parallel plate method. The present study uses the thermal property analyzer (TEMPOS, Meter, Pullman, WA, USA) with a 60 mm KS-3 sensor and a thermal bath. The KS-3 sensor is capable of measuring thermal conductivity in the range of 0.02–2.00 W/m·k with an accuracy of ±10%. The thermal conductivities of soybean oil, coconut oil, palm oil and their hybrid nanofluids with Al_2_O_3_-TiO_2_ nanoparticles were measured at three different temperatures of 30, 45 and 60 °C. The testing methodology was according to the ASTM D7896-14 standard and was similar to the procedure performed by Chiam et al. [33]. To ensure the readings of the sensors were accurate and according to the standard, a thermal conductivity measurement was first conducted on a glycerin solution with a manufacturer’s validated thermal conductivity value of 0.286 W/m·k. A total of 5 measurements were performed for each nanofluid sample and averaged for analysis. In order to minimize the measurement error due to free convection effects at the measurement sensor, a time interval of 15–20 min was maintained between each data measurement.

### 2.5. Dynamic Viscosity Measurement

The dynamic viscosity of soybean oil, coconut oil, palm oil and their hybrids with Al_2_O_3_-TiO_2_ suspensions were measured using an Anton Paar rheometer made in Graz, Austria. The rheometer was connected to a temperature control thermal bath to vary the liquid temperature during each data measurement. The dynamic viscosity measurement was performed at three different temperatures: 30, 45, and 60 °C. A minimum of 12 mL working fluid is required for the measurement of viscosity with the Anton Paar rheometer for shear rates between 0 and 100 s^−1^. The motor torque was adjusted with a quick air check, and the rheometer was further calibrated by measuring the viscosity of water and comparing it with the standard data to ensure accurate data measurement. The standard deviation and accuracy of the viscosity measurement for water were found to be less than 4%. According to Shah et al. [25], a maximum error of 3–5% is highly acceptable for rheological analysis. The shear rate and shear stress data for all base oils and their hybrids were recorded for the fluid behavior analysis.

### 2.6. Density Measurement

**Figure 1:**
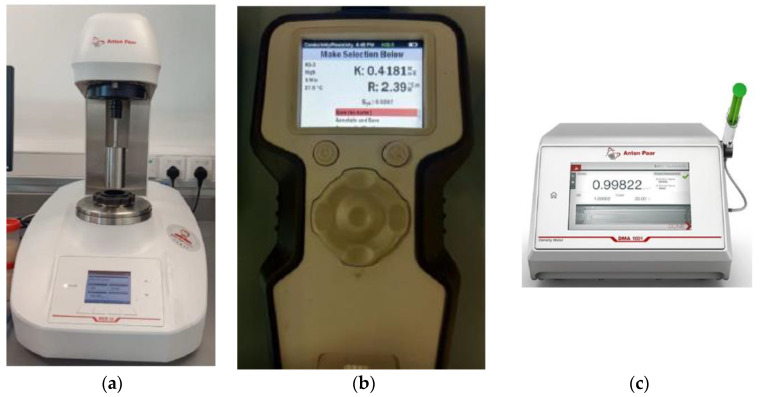
(**a**) Anton Paar rheometer, (**b**) TEMPOS, (**c**) Anton-Paar GmbH density meter.

The Anton-Paar GmbH (Graz, Austria) density meter was used to measure the density of the palm oil, coconut oil and soybean oil suspended with Al_2_O_3_-TiO_2_ nanoparticles. Before commencing the density measurement for nanofluids, the accuracy of the density meter was calibrated with air and deionized water according to the procedure stated in the user manual. The accuracy of the instrument is ±0.0001 g/cm^3^. For each measurement, a 10 mL fluid sample was loaded into the machine using a syringe, and the density measurement displayed was recorded. Similarly, the density measurement was performed at three different temperatures of 30, 45, and 60 °C. Figure 1 shows the instrument used in the experimental procedure to evaluate the thermal conductivity, viscosity, and density of the base oils and their hybrid nanofluids.

## 3. Results and Discussion

### 3.1. Stability of Nanofluids with Different Types of Vegetable Base Oil

**Figure 2:**
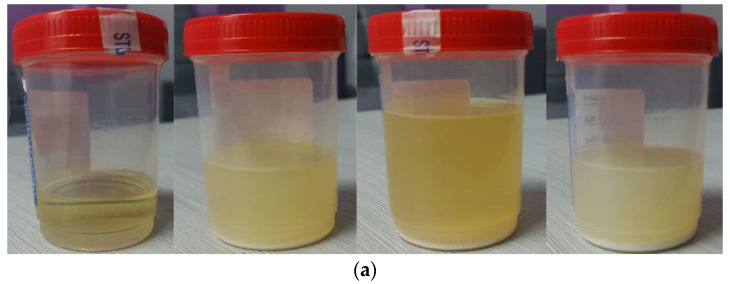

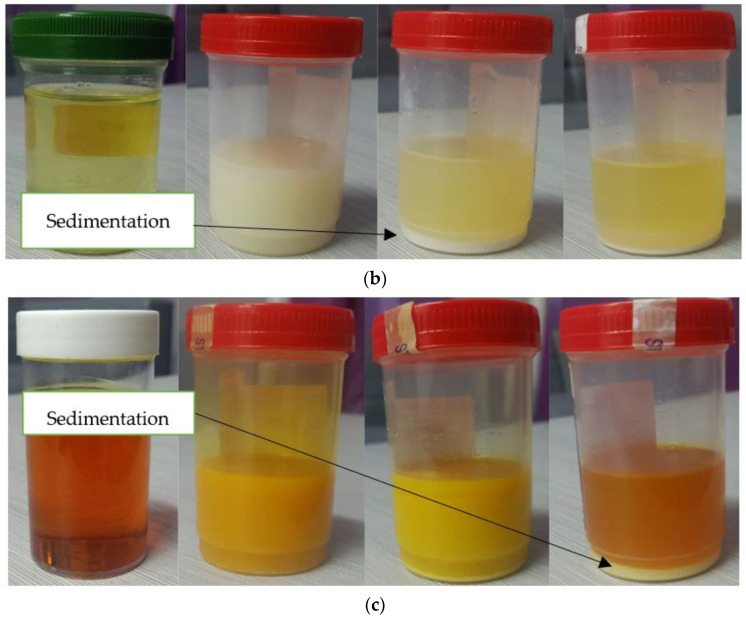
Condition of vegetable oil hybrid nanofluids after 30 days: (**a**) coconut oil, (**b**) soybean oil, and (**c**) palm oil.

The stability of soybean oil, coconut oil, and palm oil with Al_2_O_3_-TiO_2_ nanoparticles at 0.2, 0.4 and 0.6% mass concentrations were analyzed visually by the sedimentation technique. The stability of hybrid nanofluids are affected by the Van Der Waals forces of attraction between dispersed nanoparticles that agglomerate in the base oils. Figure 2 displays the condition of all the nanofluids after 30 days. As shown in Figure 2, an increase in Al_2_O_3_-TiO_2_ concentrations in the base oils created significant sedimentation of nanoparticles and a separation layer between the nanoparticles and the base oil. Among the three different vegetable oils, soybean oil and palm oil with a 0.2% particle suspension were found to be the most stable samples, with no visual sedimentation observed. The thickest sedimentation layer was observed for the palm oil nanofluid with the Al_2_O_3_-TiO_2_ hybrid at 0.6% mass concentration. The thick layer of nanoparticle sedimentation indicated agglomeration of nanoparticles and poor stability. The agglomeration of nanoparticles is due to the larger particle size of TiO_2_ nanoparticles compared with Al_2_O_3_ nanoparticles.

### 3.2. Charcterization of Nanoparticles Using XRD and TEM

**Figure 3:**
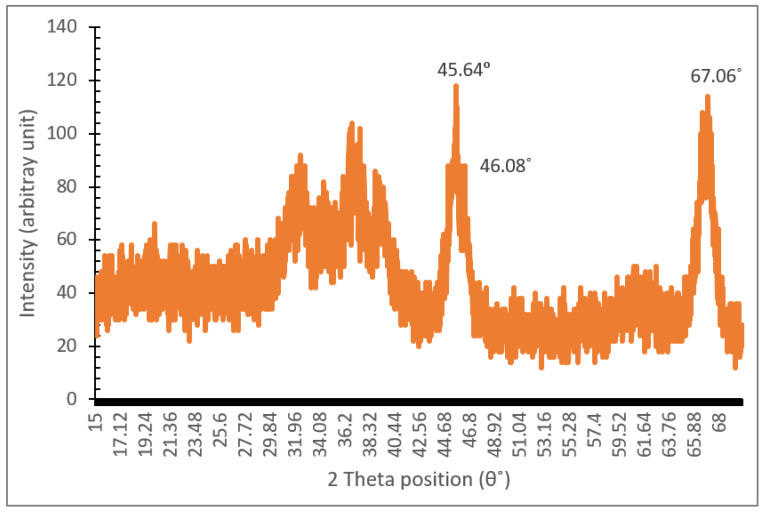
XRD spectra of Al_2_O_3_ nanoparticles.

**Figure 4:**
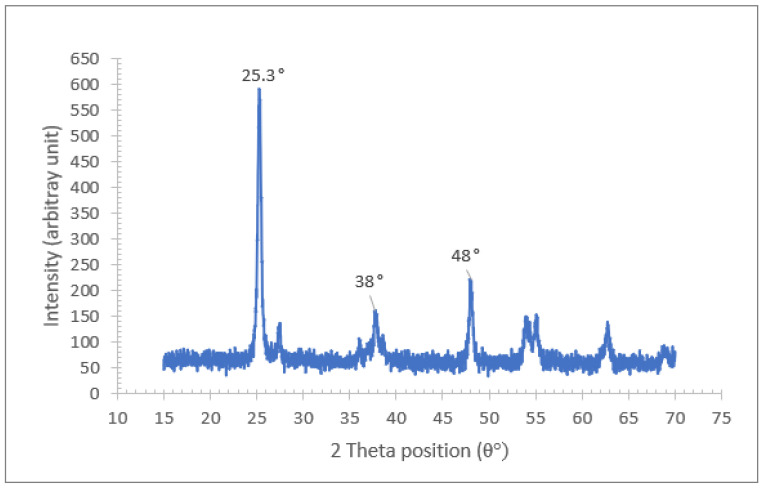
XRD spectra of TiO_2_ nanoparticles.

**Figure 5:**
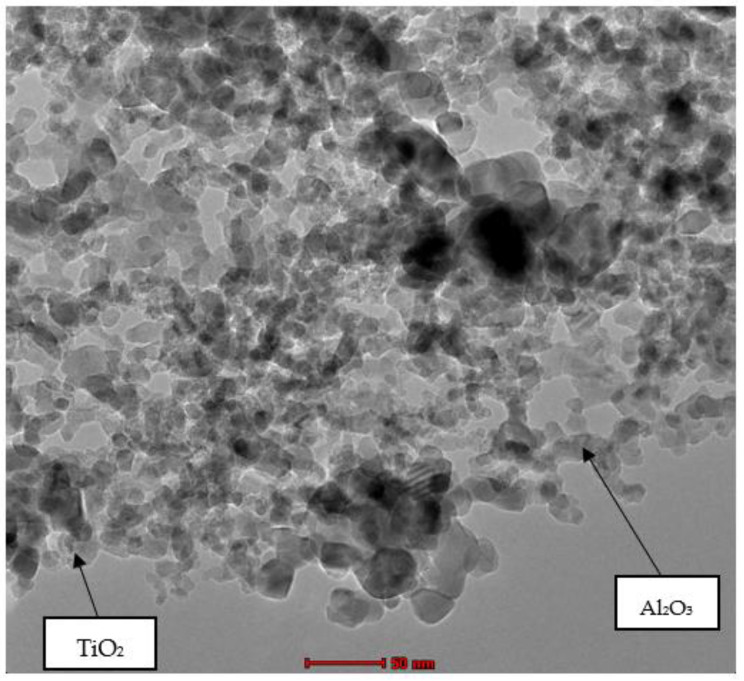
TEM image of Al_2_O_3_-TiO_2_ nanoparticles.

Two types of nanoparticles were used in the experiment: Al_2_O_3_ and TiO_2_. Figure 3 illustrates the XRD spectra of the Al_2_O_3_ nanoparticles for diffraction angles between 15° and 70° and a step interval of 0.02°. The comparison of the XRD data from the present study with the standard for ϒ-Al_2_O_3_ structure JCPDS 00-005-0661 from the International Centre for Diffraction Data (ICDD) database proves that the Al_2_O_3_ nanoparticles are crystalline in nature with a monoclinic structure. Furthermore, the wide diffraction peaks shown in Figure 3 demonstrate the small crystallite size of the Al_2_O_3_ nanoparticles. As for the XRD spectra for the TiO_2_ nanoparticles shown in Figure 4, a comparison with JCPDS card no. 21-1272 points to the anatase phase of TiO_2._ Detailed observations by Theivasanthi et al. [37] reported strong diffraction peaks at 2θ angle of 25.3°and 48.01°, corresponding to the same anatase phase. The three most dominant peaks from both XRD data were used to calculate the crystallite size of the Al_2_O_3_ and TiO_2_ nanoparticles using the Scherrer formulae. The average crystallite size calculated for the Al_2_O_3_ and TiO_2_ nanoparticles was approximately 8 nm and 19 nm, respectively. This is consistent with the particle size observed based on the TEM image of the Al_2_O_3_-TiO_2_ nanoparticle displayed in Figure 5.

Figure 5 shows the TEM image of the nanofluid suspended with Al_2_O_3_ and TiO_2_ nanoparticles. The average particle size of the TiO_2_ observed in the TEM image is about 21 nm, and the particle size of Al_2_O_3_ is approximately 8 nm. This finding is consistent with the particle size computed using the XRD data. The TEM image also shows that the smaller Al_2_O_3_ nanoparticles tend to form a chain-like structure around the much larger sized TiO_2_ nanoparticles in a condition with minimal overlapping. This clearly shows that the soybean oil and palm oil suspended with 0.2% Al_2_O_3_-TiO_2_ nanoparticles has the highest stability compared with the coconut oil and palm oil base fluids, as shown in Figure 2 in Section 3.1.

### 3.3. Thermal Conductivity of Base Oils and Their Hybrid

**Figure 6:**
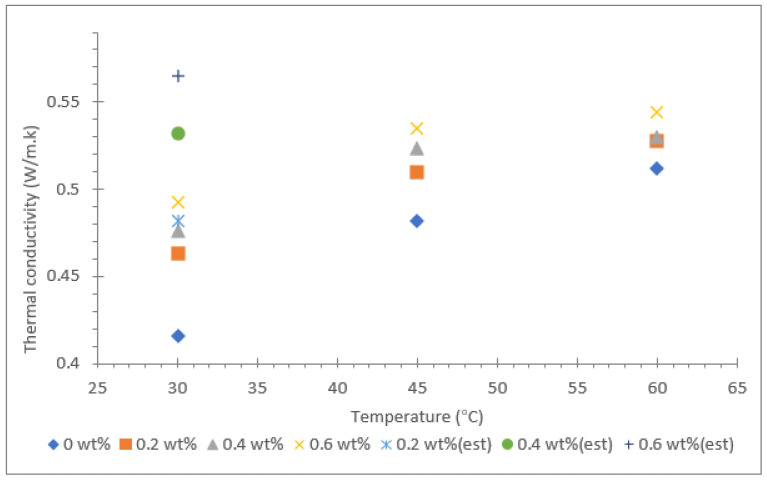
Thermal conductivity of soybean oil/Al_2_O_3_-TiO_2_ hybrid nanofluid.

**Figure 7:**
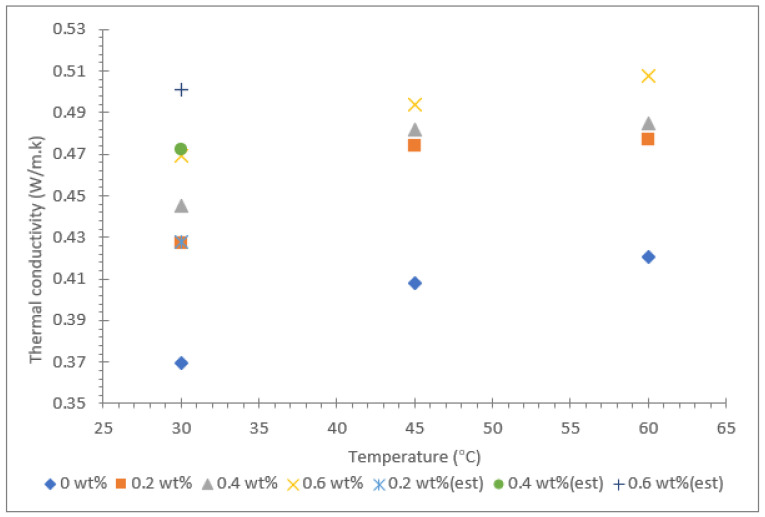
Thermal conductivity of coconut oil/Al_2_O_3_-TiO_2_ hybrid nanofluid.

**Figure 8:**
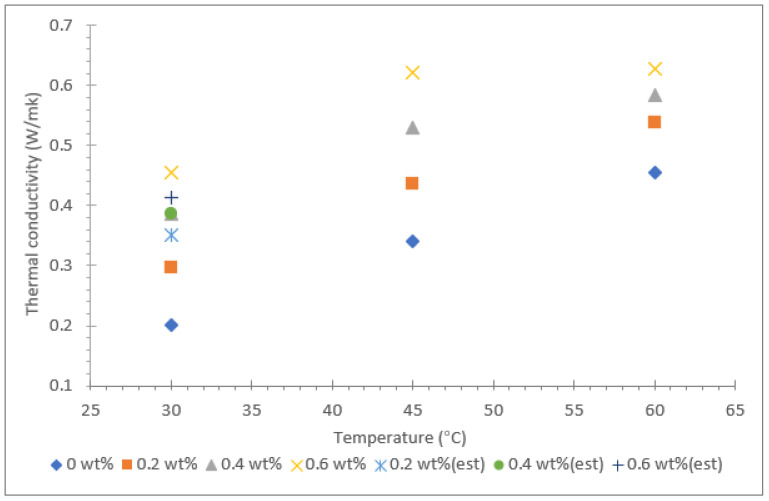
Thermal conductivity of palm oil/Al_2_O_3_-TiO_2_ hybrid nanofluid.

The thermal conductivity results for Al_2_O_3_-TiO_2_ hybrid nanofluid with soybean oil, coconut oil, and palm oil as base fluids are shown in Figure 6, Figure 7 and Figure 8. The results are based on three different temperatures of 30, 45, and 60 °C and nanoparticle mass concentrations between 0.2 and 0.6%. The thermal conductivities of all three base oils were measured and are represented with a mass concentration of 0% in the same graph displayed in Figure 6, Figure 7 and Figure 8. In addition, the theoretical Maxwell model shown in Equation (1) was used to estimate the thermal conductivity at room temperature (≈30 °C). The theoretical data for all nanofluids were fitted in the same graphs, which are shown in Figure 6, Figure 7 and Figure 8.

(1) $$k_{nf}=\frac{k_{p}+2k_{f}+2\phi \left(k_{p}-k_{f}\right)}{k_{p}+2k_{f}-\phi \left(k_{p}-k_{f}\right)}$$

Among the three vegetable oils, palm oil had the lowest thermal conductivity value of 0.202 W/m·k at 30 °C. However, as the fluid temperature was raised to 60 °C, the thermal conductivity of palm oil increased to 0.455 W/m·k, equivalent to a 125.3% increase. As for the soybean oil and coconut oil, the thermal conductivity values measured at 30 ºC were 0.426 W/m·k and 0.369 W/m·k, respectively. The enhancement of thermal conductivity for soybean oil and coconut oil due to temperature rising from 30 to 60 °C was 23.3% and 14.1%, respectively. This clearly shows that the influence of temperature in the augmentation of thermal conductivity of the base fluid is greatest for palm oil.

For the hybrid nanofluid with different vegetable base oils, the Al_2_O_3_-TiO_2_ nanoparticles resulted in a positive increase in thermal conductivity with the rise in mass concentration from 0.2 to 0.6%. The effect was consistent for all the hybrid nanofluids with soybean oil, coconut oil, and palm oil as base fluids, even though the rate of increase in thermal conductivity varied. For instance, the augmentation of thermal conductivity for nanofluid due to an increase in nanoparticle concentrations has been reported by many researchers for both water and also for oil as a base fluid [38,39,40,41,42]. The increment of thermal conductivity in the oil-based suspended nanofluid is mainly due to the particle-to-particle interactions between Al_2_O_3_ and TiO_2_ nanoparticles at higher mass proportions. Furthermore, at higher temperatures, the intensity of the Brownian motion effect is stronger, causing an increase in the thermal conductivity of oil-based hybrid nanofluids. This is consistent with the findings reported by Yuan et al. [43], in which the thermal conductivity of an MoS_2_/BA350 oil nanofluid was enhanced by 38.7% with 1.0% nanoparticle mass concentrations. Other similar research from N.S.M Sahid et al. [41] demonstrated that at high temperatures, the collision of particles occurs at a higher rate; thus, they carry more kinetic energy.

Among the three types of vegetable oil hybrids, the palm oil-based Al_2_O_3_-TiO_2_ hybrid nanofluids displayed the largest increase in thermal conductivity with the rise in nanoparticle mass concentrations from 0.2 to 0.6%. The suspension of 0.6% of Al_2_O_3_-TiO_2_ nanoparticles showed a 125.3% increase in thermal conductivity compared with the soybean oil and coconut oil hybrid nanofluids, which only exhibited an enhancement of 18.5% and 27.1%, respectively. Despite the fact that the palm oil base fluid had the lowest thermal conductivity of 0.202 W/m·k, the introduction of Al_2_O_3_-TiO_2_ nanoparticles increased the thermal conductivity to a maximum value of 0.455 W/m·k. This proves that the Al_2_O_3_-TiO_2_ nanoparticles interact well with the base fluid in aiding the heat conductivity, even at low temperatures. Another interesting finding observed for all the three vegetable oil-based hybrid nanofluids was that the thermal conductivity enhancement due to nanoparticle mass concentration was much lower at high temperatures (>50 °C). This finding is consistent for all three types of vegetable oils tested in the present study. This entirely contradicts the findings reported in the literature for water and ethylene glycol (EG)-based hybrid nanofluids, where the thermal conductivity enhancement is greater at high temperatures. For example, Hemmat et al. [44] reported that the maximum thermal conductivity enhancement of an EG-based hybrid nanofluid containing 1% of Fe_3_O_4_ and SWCNT nanoparticles was at a temperature of 50 °C. These researchers achieved a thermal conductivity enhancement of approximately 40% relative to the base fluid. Nevertheless, it must be taken into consideration that water or EG-based nanofluids have lower dynamic viscosity value than vegetable oil-based nanofluids. This is likely to affect the thermal conductivity augmentation of oil-based hybrid nanofluids.

### 3.4. Dynamic Viscosity and Rheological Properties of Base Oils and Their Hybrids

**Figure 9:**
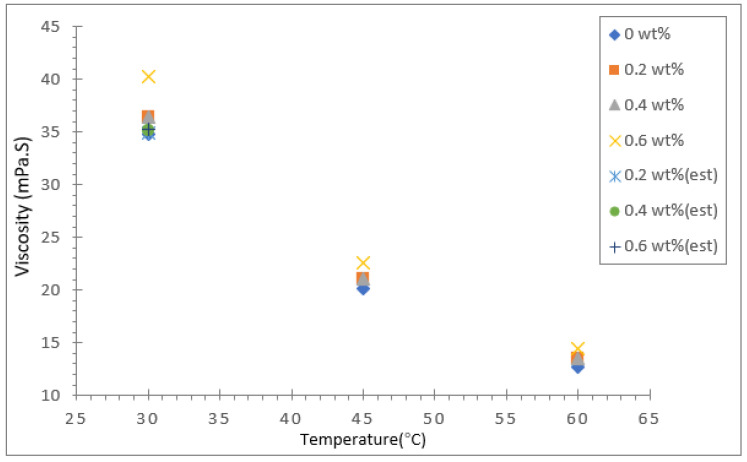
Dynamic viscosity of coconut oil/Al_2_O_3_-TiO_2._ hybrid nanofluid.

**Figure 10:**
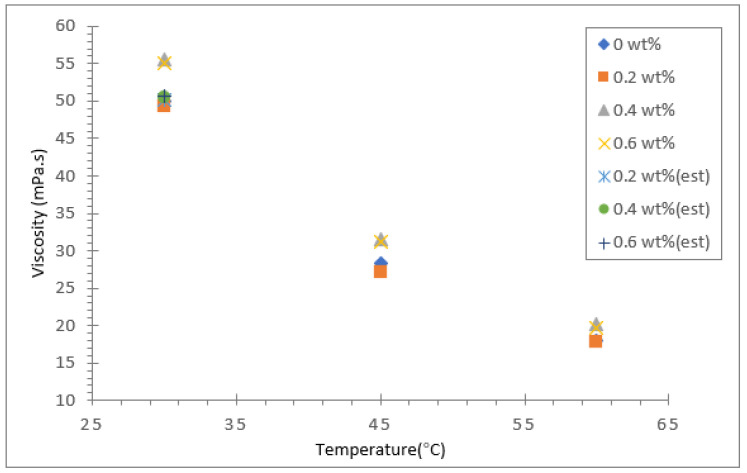
Dynamic viscosity of soybean/Al_2_O_3_-TiO_2_ hybrid nanofluid.

**Figure 11:**
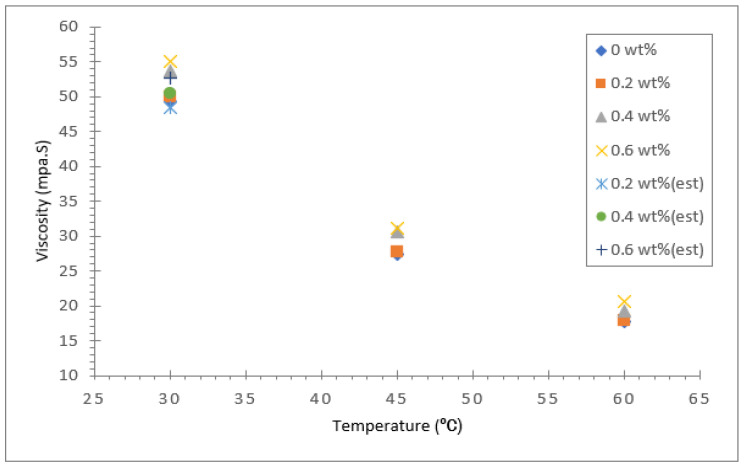
Dynamic viscosity of palm oil/Al_2_O_3_-TiO_2_ hybrid nanofluid.

The dynamic viscosity of the soybean oil, coconut oil, palm oil and their hybrid nanofluids were measured using a modular compact rheometer (Anton Paar RheoCompass) for three different temperatures: 30, 45 and 60 °C. The results for dynamic viscosity for all three vegetable base oils and their hybrids with Al_2_O_3_-TiO_2_ nanoparticle mass concentrations between 0.2 and 0.6 wt.% are presented in Figure 9, Figure 10 and Figure 11. The viscosity of the base fluid is plotted in the same graph and labelled as 0 wt.%. In addition, Brinkman’s viscosity model was used to estimate the viscosity for all three hybrid nanofluids. The computed theoretical estimated value was fitted in the same graph for the 30 °C temperature data. Among the three base oils, coconut oil was found to have the lowest viscosity value at all three temperatures investigated, followed by soybean oil and palm oil. As presented in Figure 9, the dynamic viscosity of the coconut oil-based hybrid nanofluid and base fluid was found to decrease significantly as the temperature was raised from 30 to 60 °C. For a case in point, the highest viscosity value recorded for the coconut oil-based hybrid nanofluid was about 40.22 mPa·s for a nanoparticle mass concentration of 0.6% and fluid temperature of 30 °C. However, as the temperature was raised to 60 °C, the viscosity value decreased by about 64.1% to 14.43 mPa·s. As for the soybean oil and palm oil hybrid nanofluid with an Al_2_O_3_-TiO_2_ concentration of 0.6%, the viscosity reduction due to a similar temperature rise were about 60.4% and 64.3%, respectively.

The reduction in dynamic viscosity at higher temperatures mainly results from the weakening of intermolecular strength and increased kinetic energy causing higher mobility of nanoparticles in the base fluid. This trend was also reported by Seyed et al. [45] when they experimented with the viscosity of ZnO and MoS_2_ diesel oil-based nanofluids and found that the viscosity decreased with a rise in temperature. In addition, they also noticed that the viscosity of the nanofluid was higher than the base fluid diesel at all temperatures and concentrations. In the present study, the increase in base oil viscosity due to the inclusion of nanoparticles was less significant than the effect of temperature on viscosity reduction. For coconut oil at a temperature of 30 °C, the maximum rise in viscosity was about 15.7% for an Al_2_O_3_-TiO_2_ nanoparticle concentration of 0.6%, whereas for soybean oil and palm oil, the maximum increase in viscosity was relatively low, with increases of just 6.7% and 10.9%, respectively. Moreover, the increase in viscosity of all three types of base oil with a 0.6% particle concentration and fluid temperature of 60 °C was significantly smaller. The smallest viscosity increase was observed for the soybean oil-based hybrid nanofluid. In all cases investigated, the viscosity increase due to Al_2_O_3_-TiO_2_ nanoparticles was suppressed at high temperatures (>50 °C). The maximum differences in viscosity values between the base oils and hybrid nanofluids were less than 10%.

The increase in viscosity of the base oil with the dispersion of nanoparticles in the base fluid is due to the increase in the fluid internal shear stress. The less significant effect of Al_2_O_3_-TiO_2_ nanoparticles in the vegetable base oil is a positive outcome of the study. This is because the viscosity properties of all three vegetable oils were not affected to a great extent in the hybrids. These oils could still maintain their base oil properties even though they contained nanoparticles. It is evident in Figure 9, Figure 10 and Figure 11 that as the nanofluid temperature increased to 60 °C, the nanoparticles’ effect on the dynamic viscosity decreased. One of the reasons that the inclusion of nanoparticles has less impact on the viscosity of vegetable oil compared to water or EG is that vegetable oil has a much higher intermolecular strength between the molecules [46]. The small sized Al_2_O_3_-TiO_2_ nanoparticles do not have much effect on weakening the strong bond. The limited increases in the viscosity properties of base oils widen the potential industrial application of vegetable oil-based hybrid nanofluids as insulation oil in power transformers.

**Figure 12:**
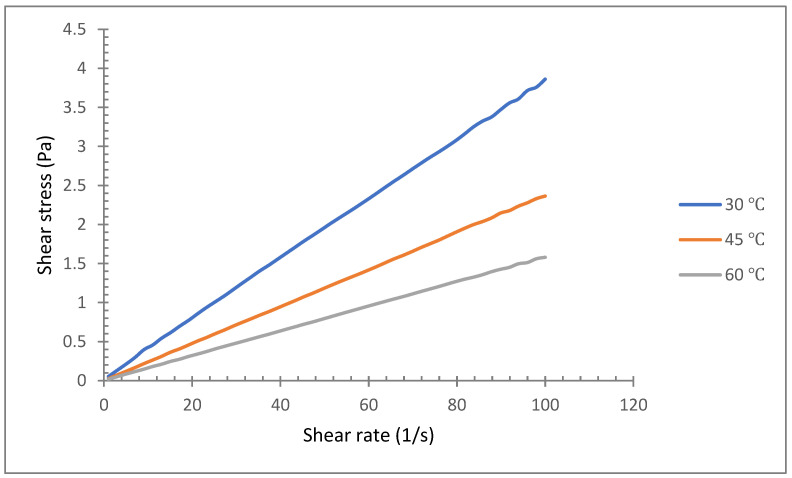
Rheological behavior of soybean oil/Al_2_O_3_-TiO_2_ hybrid nanofluid (0.2%).

**Figure 13:**
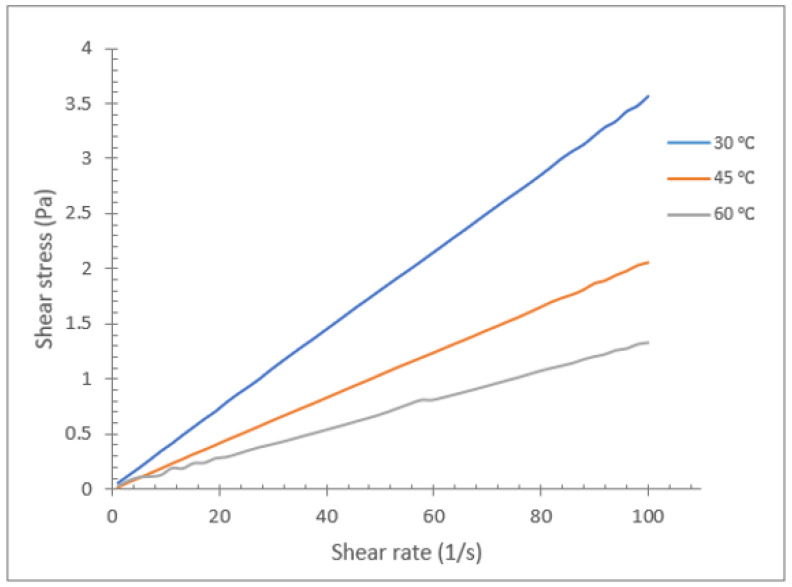
Rheological behavior of coconut oil/Al_2_O_3_-TiO_2_ hybrid nanofluid (0.2%).

**Figure 14:**
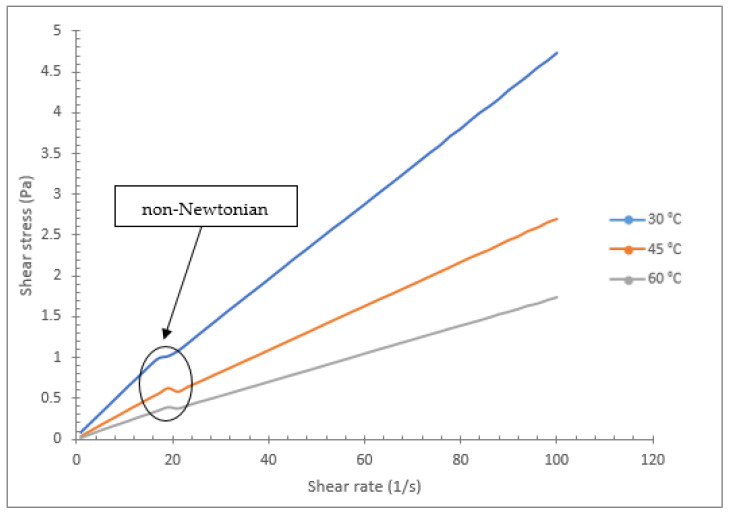
Rheological behaviour of palm oil/Al_2_O_3_-TiO_2_ hybrid nanofluid (0.2%).

The rheological characteristics of various vegetable oil-based hybrids with Al_2_O_3_-TiO_2_ nanoparticles are shown in Figure 12, Figure 13 and Figure 14. The graph in Figure 12 shows the relationship between the shear rate (s^−1^) and shear stress (Pa) for soybean oil with Al_2_O_3_-TiO_2_ at a mass concentration of 0.2% at temperatures of 30, 45, and 60 °C. The range for the shear rate is between 1 and 100 s^−1^. Theoretically, the behaviour of a fluid can be categorized as Newtonian or non-Newtonian based on the fluid flow behaviour. For instance, if the viscosity of a liquid remains constant relative to the shear rate, it is said to be a Newtonian fluid. Otherwise, if the fluid’s viscosity varies with respect to the shear rate, then it is considered a non-Newtonian fluid. This theory concerning nanofluids has been investigated by many researchers [46,47,48,49,50]. In the present study, as shown in Figure 12, Figure 13 and Figure 14, the shear stress of a vegetable oil-based nanofluid increased linearly with the shear rate for all base oils and nanoparticle concentrations. The linear relationship proves that the vegetable oil still exhibits Newtonian characteristics, even with the dispersion of Al_2_O_3_ and TiO_2_ nanoparticles. As shown in Figure 12, the gradient of the plot decreases with the increase in temperature. This is due to the reduction in viscosity values at a higher temperature gradient. For the soybean oil-based hybrid nanofluid, the lowest gradient observed is for nanofluid at a temperature of 60 °C, which represents the lowest viscosity value.

Figure 13 and Figure 14 show the rheological behaviour of the coconut oil and palm oil hybrid nanofluids. Both these vegetable oil-based hybrid nanofluids exhibit linear relationships similar to that of soybean oil. The shear stress increased linearly with the shear rate. However, for the coconut oil graph in Figure 13, the plot for the temperature of 60 °C shows a slight distortion in the shear stress for shear rates between 5 and 20 s^−1^. This variation is relatively small and has less effect on the overall Newtonian behaviour of the coconut oil nanofluid. The slight non-Newtonian behaviour of coconut oil hybrid nanofluid at low shear rates is due to the realignment of its molecular structure as the rheometer spindle is rotated at low speed. A similar finding was also reported by Sujith et al. [51] for Al_2_O_3_-coconut oil mono nanofluids. Meanwhile, Sajeeb et al. [52] also encountered similar results for shear rates between 6 and 15 s^−1^ when they tested the viscosity of coconut oil suspended with CeO_2_-CuO nanoparticles at concentrations between 0 and 1%. Consequently, their coconut oil hybrid nanofluid started behaving as a Newtonian fluid for shear rates above 15 s^−1^. The rheological results for the palm oil-based hybrid nanofluid shown in Figure 14 also indicate a variation in the viscosity at low shear rates between 19 and 23 s^−1^. The variation in viscosity indicates a non-Newtonian characteristic. However, as the shear rate increases, the fluid’s behaviour changes to Newtonian fluid characteristics. The aggregation of particles at low shear rates resulted in inconsistent viscosity values. The fluid behaved in a non-Newtonian way due to less shear thinning with the application of shear on it. Nevertheless, the non-Newtonian period is relatively short, contributing about 6% of the shear rate variation while the remaining 94% shows Newtonian behaviour.

### 3.5. Density of Vegetable Oil and Their Hybrids

**Figure 15:**
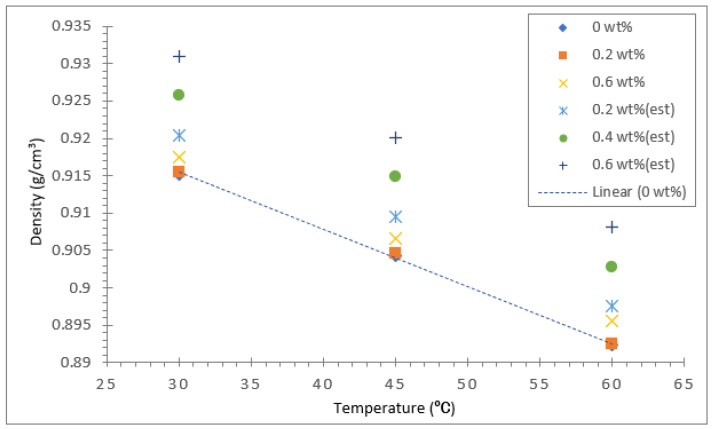
Density of coconut oil/Al_2_O_3_-TiO_2_ hybrid nanofluid.

**Figure 16:**
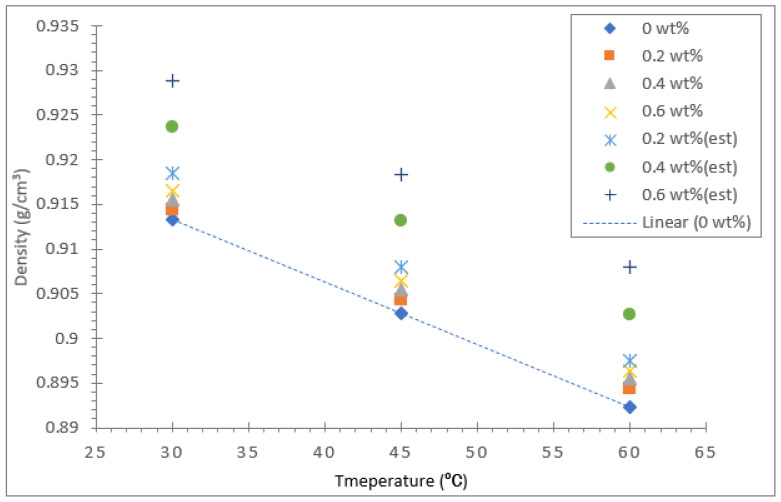
Density of soybean oil/Al_2_O_3_-TiO_2_ hybrid nanofluid.

**Figure 17:**
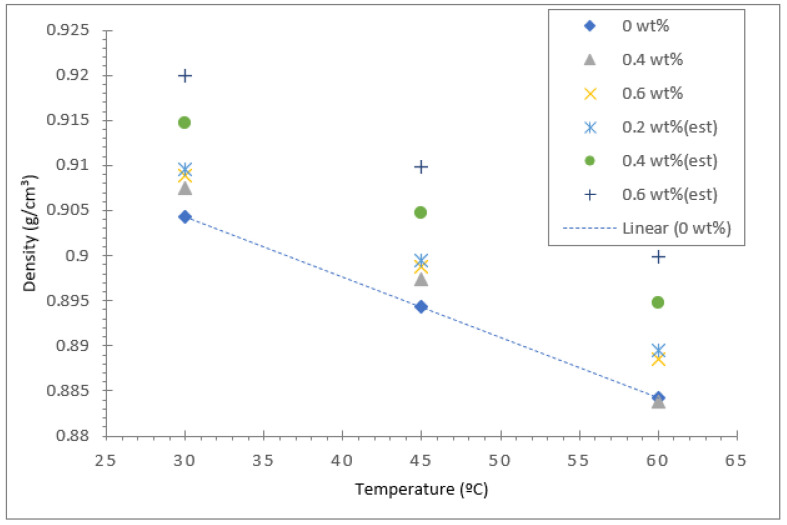
This Density of palm oil/Al_2_O_3_-TiO_2_ hybrid nanofluid.

The density of all three types of vegetable oil-based nanofluids and their base oils were measured using a density meter (Anton Paar GmbH, Graz, Austria) for three different temperatures: 30, 45, and 60 °C. Figure 15 displays the density of coconut oil and its hybrid nanofluid for concentrations between 0.2 and 0.6%. The density of the coconut base oil and the hybrid nanofluids decreased significantly with the rise in temperature from 30 to 60 °C. As for the base oil, the decrease in density with temperature shows a linear relationship, as evident in the trend line shown in Figure 15, Figure 16 and Figure 17. For validation purposes, the theoretical density values of all the nanofluids at different concentrations were calculated using the model equation from Pak and Cho [53] shown in Equation (2). The computed data were plotted in the density graph for all three different base oils, as shown in Figure 15, Figure 16 and Figure 17.

(2) $$\rho_{nf}=\rho_{p}+\left(1-\phi \right)\rho_{f}$$

The temperature rise had a significant effect on the density compared with the increase in Al_2_O_3_-TiO_2_ concentrations in coconut oil. At all three temperatures investigated, the density of the base coconut oil was lower than its hybrid nanofluid. The increase in Al_2_O_3_-TiO_2_ nanoparticles concentration raised the density of coconut oil. However, this increment was relatively small. For example, the density of coconut oil at 30 °C was about 0.91523 g/cm^3^. Meanwhile, the density of the coconut oil hybrid nanofluid with a 0.6% nanoparticle concentration was about 0.91746 g/cm^3^, which corresponds to a 0.22% increase in density compared with the base oil. On the other hand, the decrease in density of the coconut oil hybrid nanofluid with a 0.6% nanoparticle concentration due to an increase in temperature from 30 to 60 °C was about 2.4%. Therefore, the effect of temperature in density augmentation was more significant than Al_2_O_3_-TiO_2_ nanoparticle concentrations for the coconut oil-based hybrid nanofluid. The main reason for the reduction in density is the thermal expansion of oil due to an increase in temperature. A report by Muhammad Rafiq et al. [54] concluded that the density decreases as temperature rises due to thermal expansion of oil as the increase in temperature leads to expansion of liquid volume, causing its density to diminish.

Figure 16 illustrates the density results for the soybean oil base fluid and hybrid nanofluid. Similar to coconut oil, the density of soybean oil with Al_2_O_3_-TiO_2_ nanoparticles is higher than that of the base oil. The highest density value of 0.91649 g/cm^3^ was recorded for the soybean oil hybrid nanofluid with a 0.6% nanoparticle concentration at a liquid temperature of 30 °C. In contrast, the lowest density value of 0.8933 g/cm^3^ was measured for the base soybean oil at 60 °C. The increase in nanoparticle concentration from 0.2 to 0.6% in soybean oil caused an increase in the density of the base oil of at most 0.35%, which is larger than the 0.13% increment observed for coconut oil. The effect of temperature on density augmentation was more significant for both the base oil and the hybrid soybean nanofluid, and it displayed an almost a linear decline as the temperature is varied from 30 to 60 °C. The maximum decrease in density for the soybean hybrid nanofluid was about 2.24%, slightly lower than the decline recorded for the coconut oil hybrid displayed in Figure 15. This finding is consistent for the base fluid because the coconut oil base fluid had a density reduction of 2.5% compared with soybean oil, which had a density reduction of 2.29%. Therefore, the density reduction for their hybrids follows the same trend as the base fluids since the effect of nanoparticle concentration is less significant compared with the effect of temperature.

Among the three vegetable oils tested, palm oil had the lowest viscosity value at all temperatures investigated. This is based on the density graph presented in Figure 17. The density of the palm oil base fluid without any nanoparticle suspension was measured to be 0.90431 g/cm^3^, 0.89427 g/cm^3^, and 0.88423 g/cm^3^ for 30, 45, and 60 °C, respectively. The measured density data for the palm oil-based hybrid nanofluids demonstrates a similar trend in density augmentation as the coconut oil and soybean oil hybrid nanofluids. In addition, the increase in density of palm oil due to Al_2_O_3_-TiO_2_ nanoparticle concentrations is noted to be the highest compared with the other two base oils, with a 0.5% enhancement in density. This shows an increased effect of nanoparticle concentration in density enhancement when using palm oil as the base fluid.

### 3.6. Experipental Uncertainty and Error Analysis

The repeated experimental data were used to calculate the relative uncertainty for the thermal conductivity, dynamic viscosity, and density measurements. The highest relative uncertainty percentage for each thermophysical property is taken for comparison purposes. The fractional uncertainty value is calculated based on the average deviation divided by the average value. The highest uncertainty percentage was obtained for coconut oil-based hybrid nanofluid thermal conductivity measurements with a maximum uncertainty of 0.97%. The lowest percentage uncertainty of 0.009% was obtained for the measurement of the density of palm oil-based hybrid nanofluid. For the density measurements, the uncertainty in experimental data was relatively low for all types of base oil and their hybrids. A low uncertainty value indicates a very high precision in the data measurement. As for the dynamic viscosity data, the uncertainty is less than 0.08%, reflecting a low ratio of the absolute uncertainty to the reported value. The standard error was computed based on the five measurements recorded for each sample. The lowest standard error was observed for the density data, with a standard error of 0.05% and a standard deviation of 0.000111. The highest standard error was computed for the thermal conductivity data of the hybrid nanofluids with a maximum error of 0.26% and sample standard deviation of 0.00458. Meanwhile, for the dynamic viscosity data measurement, the highest standard error was about 0.34%. The low standard errors for all the experimental data indicates a low amount of discrepancy in the measurements.

## 4. Conclusions

In the present study, experimental work was carried out to evaluate the thermophysical properties of vegetable oil suspended with Al_2_O_3_-TiO_2_ nanoparticles at mass concentrations of 0.2, 0.4, and 0.6%. The characterization of the Al_2_O_3_ and TiO_2_ nanoparticles indicated that the sizes of the nanoparticles are within the range specified in the material datasheet provided by Sigma Aldrich. The average measured size of the Al_2_O_3_ nanoparticles was about 8 nm and for TiO_2_ it was about 19 nm. Among the three base oils, it was concluded that soybean oil has the highest thermal conductivity, followed by palm oil and then coconut oil. The experimental results indicate that the thermal conductivity of both the base fluids and their hybrids increases with temperature and nanoparticle concentrations for all three types of base oils. The palm oil-based hybrid nanofluid with 0.6% a Al_2_O_3_-TiO_2_ concentration has the highest thermal conductivity enhancement of 125.3%, demonstrating good compatibility of the Al_2_O_3_-TiO_2_ nanoparticles with the base fluid. Therefore, the Al_2_O_3_-TiO_2_ nanoparticles have a more significant effect on thermal conductivity enhancement in palm oil than in soybean oil and coconut oil. Good thermal conductivity enhancement is important in aiding heat transfer for transformer cooling applications. In all base oils, increased nanoparticle concentrations resulted in a slight increase in the viscosity of the base oils. This increment, however, was suppressed by the effect of temperature as the viscosity of the hybrid nanofluids for all the base fluids and concentrations decreased significantly with temperature rises. The lowest viscosity value was observed for the coconut oil-based Al_2_O_3_-TiO_2_ hybrid nanofluid with a 0.2% nanoparticle concentration at 60 °C. The effect of nanoparticle concentration on viscosity increments was less than 10% for all the base oils. In addition, the suspension of Al_2_O_3_-TiO_2_ nanoparticles in the base oils did not change the Newtonian fluid characteristics of the base oils except for the palm oil-based hybrid nanofluid, which behaved as a non-Newtonian fluid at low shear rates. Based on the density results, it can be concluded that the rise in temperature caused a reduction in the density for all three types of hybrid nanofluids at nanoparticle concentrations between 0.2 and 0.6%. Palm oil had the lowest density value compared with soybean oil and coconut oil. The increase in density due to the increase in Al_2_O_3_-TiO_2_ nanoparticles was more significant for palm oil than for coconut oil and soybean oil. Meanwhile, the influence of temperature on density reduction was more significant for the coconut oil and soybean oil-based Al_2_O_3_-TiO_2_ hybrid nanofluids. Overall, the palm oil-based hybrid nanofluid has better thermophysical properties compared with soybean oil and coconut oil. Furthermore, this study provides valuable knowledge on the effect of hybridization of Al_2_O_3_-TiO_2_ nanoparticles in vegetable oils such as coconut oil, palm oil, and soybean oil. The results demonstrate the bright potential of palm oil-based hybrid nanofluid as an alternative for transformer oil. Further research work on the electrical and dielectric properties is required to determine the significance of vegetable oil-based hybrid nanofluids as insulation oil in power transformers.

## Figures and Tables

**Table 1:** Physical properties of Al_2_O_3_ and TiO_2_ nanoparticles [34].

Properties	Nanoparticles	
	Al_2_O_3_	TiO_2_
Colour	White	White
Size	<13 nm	<21 nm
Purity	99.8%	99.5%
Morphology	Spherical	Spherical

**Table 2:** Properties of vegetable oil.

Properties	Coconut Oil	Soybean Oil	Palm Oil
Dielectric strength (kV)	60	39	25
Viscosity(mPa·s at 40 °C)	29	34.5	29.2
Density (g/cm^3^)	0.917	0.9	0.9
Pour point (°C)	23	−1	15
Flash point (°C)	225	234	242

## Data Availability

Not applicable here.
